# Automatic quality control of single-cell and single-nucleus RNA-seq using valiDrops

**DOI:** 10.1093/nargab/lqad101

**Published:** 2023-11-18

**Authors:** Gabija Kavaliauskaite, Jesper Grud Skat Madsen

**Affiliations:** Department of Biochemistry and Molecular Biology, University of Southern Denmark, Odense M 5230, Denmark; Center for Functional Genomics and Tissue Plasticity (ATLAS), Odense M 5230, Denmark; Center for Functional Genomics and Tissue Plasticity (ATLAS), Odense M 5230, Denmark; Department of Mathematics and Computer Science, University of Southern Denmark, Odense M 5230, Denmark; The Novo Nordisk Foundation Center for Genomic Mechanisms of Disease, Broad Institute of MIT and Harvard, Cambridge, MA 02142, USA

## Abstract

Single-cell and single-nucleus RNA-sequencing (sxRNA-seq) measures gene expression in individual cells or nuclei enabling comprehensive characterization of cell types and states. However, isolation of cells or nuclei for sxRNA-seq releases contaminating RNA, which can distort biological signals, through, for example, cell damage and transcript leakage. Thus, identifying barcodes containing high-quality cells or nuclei is a critical analytical step in the processing of sxRNA-seq data. Here, we present valiDrops, an automated method to identify high-quality barcodes and flag dead cells. In valiDrops, barcodes are initially filtered using data-adaptive thresholding on community-standard quality metrics, and subsequently, valiDrops uses a novel clustering-based approach to identify barcodes with distinct biological signals. We benchmark valiDrops and show that biological signals from cell types and states are more distinct, easier to separate and more consistent after filtering by valiDrops compared to existing tools. Finally, we show that valiDrops can predict and flag dead cells with high accuracy. This novel classifier can further improve data quality or be used to identify dead cells to interrogate the biology of cell death. Thus, valiDrops is an effective and easy-to-use method to improve data quality and biological interpretation. Our method is openly available as an R package at www.github.com/madsen-lab/valiDrops.

## Introduction

The widespread adaptation of single-cell and single-nucleus RNA-sequencing (sxRNA-seq) is producing new and revolutionizing insights into the function of cells and tissues. However, during the isolation of cells or nuclei for sxRNA-seq, they can become apoptotic, stressed or damaged. The magnitude of these artefacts is protocol-specific and can lead to distortion of the biological signal, for example, through activation of early response factors, induction of apoptosis and transcript leakage (1–4). In addition to distorting the biological signal, these processes also lead to the contamination of the solution with debris, such as cell-free ambient RNA (5,6). This problem is exacerbated when processing solid tissues, where harsh methods can be required to release single cells, or when processing nuclei, where cells are lysed, and their contents are released.

Currently, most high-throughput sxRNA-seq methods either use combinatorial indexing [e.g. sci-RNA-seq (7), SPLiT-seq (8) and scifi (9)] or are based on droplet emulsions [e.g. Chromium Single Cell Gene Expression (10), Drop-seq (11), inDrop (12) or HyDrop (13)]. In droplet-based methods, microfluidics is used to process single cells or nuclei in a water-in-oil emulsion, which contains the necessary reagents to synthesize complementary DNA with droplet-specific barcodes. Debris may be captured together with cells or nuclei adding noise to the biological signal, or debris may be encapsulated into empty droplets creating unwanted signals. Thus, the isolation process can introduce at least three analytical challenges: to separate contaminated empty droplets from cell- or nucleus-containing droplets; to separate cell- or nucleus-containing droplets with a high signal-to-noise ratio from those with a low signal-to-noise ratio; and to identify droplets containing cells or nuclei, which were not strongly affected by the isolation process.

Several methods have been developed to address the first two challenges: EmptyDrops (14) fits a Dirichlet-multinomial distribution to an estimated ambient RNA and removes droplets, whose expression profile does not significantly differ from the fitted distribution. CB2 (15) extends EmptyDrops by introducing a clustering step prior to comparison with the fitted ambient RNA distribution. DIEM (16) clusters barcodes initially using *K*-means clustering on principal components (PCs) and then optimizes the cluster labels using a semi-supervised expectation–maximization algorithm. Finally, DIEM removes contaminated barcodes if they are assigned to debris clusters or have high expression of genes significantly enriched in debris clusters. Both EmptyNN (17) and CellBender (18) use deep learning models to identify and remove empty and highly contaminated barcodes. EmptyNN employs positive-unlabelled learning to directly identify which barcodes to remove, while CellBender uses an unsupervised generative model to learn the background RNA profile and recover uncontaminated counts from non-empty droplets. Finally, dropkick (19) automatically labels barcodes as informative or non-informative based on the number of detected genes and then refines labels using a logistic regression model with elastic net regularization.

Except for dropkick, all the above methods are dependent on a threshold that separates cell- or nucleus-free barcodes from putative cell- or nucleus-containing barcodes. Furthermore, except for CellBender, DIEM and dropkick, the above methods require additional filtering to remove barcodes, which contain a low-quality cell or nucleus. Commonly, this is done by thresholding the fraction of unique molecular identifiers (UMIs) derived from mitochondrial genes, the total number of detected genes and the total number of UMIs (20).

Here, we present valiDrops, an automated method for identifying cell- or nucleus-containing barcodes with a high signal-to-noise ratio and low signal distortion. Our method is open source and available as an R package at www.github.com/madsen-lab/valiDrops. We showcase how each step in valiDrops improves the data quality and we extensively benchmark valiDrops and existing methods using 47 real samples from five different studies to show that valiDrops has the best performance for both single-cell and single-nucleus RNA-seq data. Finally, we demonstrate how valiDrops, unlike existing methods, is also able to detect and flag dead cells to further increase data quality or to enable interrogation of the biological processes leading to cell death.

## Materials and methods

### Quality filtering and dead cell prediction with valiDrops

The first stage in valiDrops is to remove lowly sequenced barcodes that likely represent barcodes primarily containing ambient RNA. The log-transformed total number of UMIs as a function of the log-transformed rank in order of decreasing total UMIs (which is the basis of the so-called barcode rank plot) is smoothened using the rolling mean with a bin size defined using the Rice rule $( {b = 2\sqrt[3]{n}} )$. Piecewise linear regression is fitted to the smoothened curve using segmentation. In the default settings, models with two to five breakpoints are fitted. For each model, the root-mean-square error (RMSE) of the fit is calculated and the smallest number of breakpoints that have an error with 1.5 times the smallest RMSE is selected. The angle between all piecewise linear curves is calculated from left to right, such that negative angles correspond to the transition from a negative to a flat slope (or a flat to a positive slope, which should not occur due to ordering by rank) and positive angles correspond to the transition from a positive to a flat slope. The breakpoint with the most negative angle is selected (corresponding to the transition from the largest negative to the flattest slope) as the threshold for filtering lowly sequenced barcodes.

The second stage in valiDrops is to collect and filter on common quality metrics. To collect quality metrics, valiDrops initially automatically finds and calculates the fraction of UMIs associated with mitochondrially encoded genes (mitochondrial fraction), protein-coding genes (coding fraction) and genes encoding ribosomal genes (ribosomal fraction), as well as the log-transformed total number of UMIs and the log-transformed total number of detected genes. This automatic detection works for most common model organisms (human, mouse, rat, fly, worm and zebrafish) and common gene annotations (Ensembl, Entrez, HGNC, MGI and gene symbols), but can be overwritten by users studying other organisms or using other gene annotations. Optionally, valiDrops can calculate the fraction of UMIs associated with exons (or introns) that has been shown to be a valuable additional metric for quality filtering (21). To filter on the mitochondrial fraction, valiDrops fits a mixture of two normal distributions to the log-transformed total feature count using mixtools (22) to identify a group of high-coverage barcodes that are probably high-quality barcodes. For each putative threshold between the median mitochondrial fraction in high-coverage barcodes and one in increments of 0.001, the number of high-coverage barcodes passing the filter is calculated and the final threshold is selected by finding the knee point of the curve using inflection. In rare edge cases with generally high mitochondrial fractions, this approach identifies thresholds above 0.3, which is not biologically plausible (23). In these cases, valiDrops uses piecewise linear regression between the mitochondrial fraction and the log-transformed number of detected genes to set a stricter threshold. To filter on the coding fraction (and optionally the exon or intron fraction), valiDrops calculates the median of the fraction as well as *S*_n_, which is a more efficient alternative robust scale estimator than the median absolute deviation (24). Barcodes more than three times *S*_n_ above or below the median are removed. Finally, to filter the relationship between the log-transformed total number of UMIs and the number of detected genes, valiDrops fits a piecewise linear regression model with three breakpoints, and for each barcode calculates the residuals. Barcodes more than five times *S*_n_ above or below the median residual are removed.

The third stage in valiDrops is to collect and filter on expression-based metrics. First, 5000 highly variable genes (HVGs) are selected using scry, which are then decomposed using singular value decomposition with irlba on normalized, log-transformed and standardized counts. The PCs are clustered using Seurat (25) using resolution 0.1 (shallow clustering) and the highest resolution that does not produce any clusters containing <5 barcodes (deep clustering). Differential expression analysis is performed using presto between each deep cluster and all other barcodes that are not in the same shallow cluster as the deep cluster. To filter on expression-based metrics, valiDrops evaluates the top 10 most significantly enriched marker genes for each deep cluster. To pass filtering, none of these genes can be expressed in at least 1% fewer barcodes in the deep cluster compared to barcodes not in the deep cluster, and they must on average be expressed in >30% of the barcodes in the deep cluster, <70% of the barcodes not in the deep cluster and at least in 20% more of the barcodes in the deep cluster compared to barcodes not in the deep cluster. These filters remove deep clusters that are enriched for genes that are ubiquitously expressed. Next, valiDrops evaluates the total number of differentially expressed genes and their significance levels. To pass filtering, at least 1% of the tested genes for a deep cluster must be significant and the most significant gene must pass a data-adaptive threshold that is based on the relationship between the false discovery rate (FDR)-corrected *P*-value of the maximally significant gene in a cluster and the average difference in percent of cells expressing marker genes between the target cluster and barcodes not in the same low-resolution cluster. These filters remove deep clusters that have weak or no enrichment of specifically expressed genes. Finally, deep clusters that have a mitochondrial or ribosomal fraction higher than three times *S*_n_ above the median fraction across all clusters are removed.

The fourth stage in valiDrops is to predict dead cells. First, valiDrops arcsine-transforms the proportion of UMIs assigned to ribosomal genes (*R*) and to protein-coding genes (*C*) and rescales the transformed values to a range between 0 and 1 by dividing by half pi. The log-transformed total UMI count (*U*) and the log total number of features (*F*) are then centred, and an initial score is calculated using the following function:


\begin{eqnarray*} {\rm score} &=& \ - 11.82 U + 2.08 F + 158.98 R \nonumber\\ && \quad +\, 18.87 C F - 125.9 R C.\end{eqnarray*}

Next, initial labels are created by a data-adaptive threshold, which is defined as the first knee point in the empirical distribution function of scores between the 0% quantile and an upper quantile of 10%. Barcodes with scores below the threshold are labelled as putative dead cells, and barcodes with scores above the threshold are labelled as putative live cells. If no barcodes are labelled as putative dead by this approach, the upper quantile is increased in steps of 10%. If <3 barcodes, or >10% of barcodes, are labelled as putative dead cells, it is likely that there are no truly dead cells or that the score is likely not well calibrated for the dataset, and label optimization is halted. If no barcodes that passed quality control are labelled as putative dead, the barcode with the high score is temporarily relabelled as passing quality control. Next, the top 100 PCs are calculated using irlba based on the top 2000 HVGs found using scry. Next, labels are iteratively refined over maximally 10 epochs. Each epoch involves first selecting features using Kendall’s tau rank correlation coefficients between the PCs and the labels, and then randomly selecting labels based on their class probability from the previous epoch (initialized with equal probability), introducing random noise by jittering the features and fitting a ridge regression model using glmnet (26) using 5-fold cross-validation on the noised features and sampled labels. To increase robustness and assess the quality of the fit, 10 independent runs of label refinement are performed and only barcodes that have the same label in at least 8 runs are labelled and runs are failed if too many barcodes are not labelled.

### Benchmarking quality filtering

#### Datasets

The following public datasets were used (Supplementary Table S3):

- Single-cell RNA-seq (scRNA-seq) in human peripheral blood mononuclear cells (PBMCs): Unfiltered count matrices were downloaded from 10X Genomics.
- scRNA-seq in human lungs (27): Unfiltered count matrices from healthy donors were downloaded from NCBI Gene Expression Omnibus (GEO) under accession number GSE122960.
- scRNA-seq in human islets of Langerhans (28): Raw data were downloaded from the European Nucleotide Archive (ENA) under accession number GSE114297. Count matrices were generated using STARsolo (29), the human genome version hg38 and Ensemble gene annotations.
- Single-nucleus RNA-seq in human PBMCs: Unfiltered count matrices were downloaded from 10X Genomics.
- Single-nucleus RNA-seq in human brain (30): Raw data from healthy donors were downloaded from GEO under accession number GSE174332. Count matrices were generated using STARsolo (29), the human genome version hg38 and Ensemble gene annotations.

#### Processing

For all datasets, all methods were run with default parameters. For EmptyDrops (14) and scCB2 (15), barcodes with an FDR ≤ 0.01 were retained. For DIEM (16), calls were made using debris clusters and barcodes labelled with Clean were retained. For EmptyDrops, scCB2 and EmptyNN barcodes were also filtered on the fraction of UMIs derived from mitochondrially encoded genes using miQC (31) for scRNA-seq datasets and by removing barcodes three times the median absolute deviation above the median mitochondrial fraction for single-nucleus RNA-seq, which corresponds to a strict filtering regime (32).

#### Metrics

Labels were transferred to barcoding passing quality control using Azimuth (25) and associated reference atlases and labels (PBMCs mapped to PBMC reference: level 1: celltype.l1 labels, level 2: celltype.2; islets mapped to pancreas reference: levels 1 and 2: annotation.l1; brain mapped to motor cortex reference: level 1: subclass, level 2: cluster; and lung mapped to lung v2 (HCLA): level 1: ann_level 3, level 2: ann_finest_level). For each dataset, a total of nine metrics were calculated based on level 2 labels unless otherwise indicated:

- The average mapping score is calculated by Azimuth (25) during label transfer. Higher scores indicate better label transfer; methods were ranked in decreasing order.
- The average label score for level 2 labels is calculated by Azimuth during label transfer. Higher scores indicate better label transfer; methods were ranked in decreasing order.
- The fraction of labels that have the correct label hierarchy, which is defined as barcodes where the transferred level 1 and level 2 labels are child–parent labels in the reference atlas. Higher fractions indicate more consistent labelling across granularities; methods were ranked in decreasing order.
- The entropy of the top 200 most highly expressed genes in each label class was calculated using BioQC (33). Lower entropy indicates lower heterogeneity; methods were ranked in increasing order.
- Median local inverse Simpson’s index (LISI) (34) across transferred labels in the first 10 PCs calculated from 2000 HVGs using Seurat (25). Lower LISI indicates lower mixing of cell type labels; methods were ranked in increasing order.
- The average silhouette width across transferred cell type labels using Euclidean distances in the first 10 PCs was calculated from 2000 HVGs using Seurat (25). Higher silhouette width indicates a better separation of cell types; methods were ranked in decreasing order.
- Adjusted Rand index (ARI) was calculated using cluster labels obtained using Louvain clustering using Seurat (25) and the transferred labels. Clustering was performed with resolutions between 0.1 and 2.0 in steps of 0.1. The maximum ARI was used. High values indicate better consistency between the obtained clusters and the cell type labels; methods were ranked in decreasing order.
- Normalized mutual information (NMI) was calculated using the same strategy as for ARI. High values indicate better consistency between the obtained clusters and the cell type labels; methods were ranked in decreasing order.
- V-measure was calculated using the same strategy as for ARI. High values indicate better consistency between the obtained clusters and the cell type labels; methods were ranked in decreasing order.

An overall rank was calculated by calculating the average rank across all nine metrics and ranking the average in increasing order. A rank for label mapping was calculated using the first three metrics (mapping score, label score and label hierarchy), a rank for expression similarity corresponds to ranks of the expression entropy metrics, a rank for label cohesion was calculated using the median LISI and the average silhouette width, and a rank for label clusterability was calculated using the ARI, NMI and V-measure. Across all ranks, rank 1 corresponds to the lowest average across the ranked metrics and indicates the best performance.

For benchmarking after integration, each sample was quality controlled individually, labelled using Azimuth (25) and integrated using the RPCA method in Seurat (25) with default parameters. In the integrated space, we calculated 12 metrics associated with the quality of integration, the extent of batch effects and the consistency of biological signals using level 2 labels:

- For each label class, the fraction of marker genes, defined as differentially expressed genes [FDR ≤ 0.05, fold change ≥ 1, area under a receiver operating characteristic curve (AUC) ≥ 0.7] in a class label compared to all other barcodes, which were detected in at least three samples. Higher fractions indicate higher biological consistency between datasets; methods were ranked in decreasing order.
- The median Spearman’s rank coefficient of correlation between pseudo-bulk expression levels per class label per dataset. Higher correlations indicate higher biological consistency between datasets; methods were ranked in decreasing order.
- LISI (34) across transferred labels in the first 10 PCs in the integrated space calculated from 2000 HVGs using Seurat (25). Lower LISI indicates lower mixing of cell type labels; methods were ranked in increasing order.
- LISI (34) across dataset labels in the first 10 PCs in the integrated space calculated from 2000 HVGs using Seurat (25). Higher LISI indicates better mixing of batches; methods were ranked in decreasing order.
- The average silhouette width across transferred type labels using Euclidean distances in the first 10 PCs in the integrated space was calculated from 2000 HVGs using Seurat (25). Higher silhouette width indicates a better separation of cell types; methods were ranked in decreasing order.
- The average silhouette width across transferred dataset labels using Euclidean distances in the first 10 PCs in the integrated space was calculated from 2000 HVGs using Seurat (25). Lower silhouette width indicates less separation of batches; methods were ranked in increasing order.
- ARI was calculated using cluster labels obtained using Louvain clustering using Seurat (25) and the transferred labels. Clustering was performed with resolutions between 0.1 and 2.0 in steps of 0.1. The maximum ARI was used. High values indicate better consistency between the obtained clusters and the cell type labels; methods were ranked in decreasing order.
- ARI was calculated using cluster labels and dataset labels performed as metric 7. Low values indicate better batch integration; methods were ranked in increasing order.
- NMI was calculated using cluster labels and transferred labels performed as metric 7. High values indicate better consistency between the obtained clusters and the cell type labels; methods were ranked in decreasing order.
- NMI was calculated using cluster labels and dataset labels performed as metric 7. Low values indicate better batch integration; methods were ranked in increasing order.
- V-measure was calculated using cluster labels and transferred labels performed as metric 7. High values indicate better consistency between the obtained clusters and the cell type labels; methods were ranked in decreasing order.
- V-measure was calculated using cluster labels and dataset labels performed as metric 7. Low values indicate better batch integration; methods were ranked in increasing order.

An overall rank was calculated by calculating the average rank across all 12 metrics and ranking the average in increasing order. A rank for expression similarity was calculated based on the conservation of marker genes and Spearman’s correlation coefficient, a rank for label cohesion was calculated using the median LISI across labels and the average silhouette width for labels, a rank for label clusterability was calculated based on the ARI, NMI and V-measure for clustering labels, a rank for batch cohesion was calculated using the median LISI across batches and the average silhouette width for batches, and a rank for batch clusterability was calculated using the ARI, NMI and V-measure for clustering batches. Across all ranks, rank 1 corresponds to the lowest average across the ranked metrics and indicates the best performance.

### Evaluating the impact of each stage of valiDrops

To evaluate how each filtering stage in valiDrops affects quality metrics, we calculated the nine metrics used for method comparison using barcodes kept by stages 1, 2 and 3 in valiDrops, respectively, for all benchmarking datasets. In addition, we estimated the ambient RNA fraction using DecontX (6) defining barcodes with a total of 50 UMIs or less as background barcodes.

### Benchmarking prediction of dead cells

For the O’Flanagan datasets (3), pre-processed data for sorted dead and sorted live cells were downloaded from Zenodo under DOI: 10.5281/zenodo.3407791. For the Ordoñez-Rueda datasets (35), raw sequencing data from 10X Genomics runs were downloaded from ENA under accession number PRJEB33078 and aligned to the human genome version hg38 using STARsolo (29).

Subsequently, sorted dead and sorted live cells were quality filtered using valiDrops with modifications. In the O’Flanagan datasets, the initial step was skipped as the datasets had already been filtered. Furthermore, in both the O’Flanagan datasets (3) and the Ordoñez-Rueda datasets (35), a common threshold for mitochondrial filtering was calculated using the live cells and applied to the dead cells. After filtering, the datasets were combined, and dead cells were identified using valiDrops with default parameters. Barcodes that pass quality control were embedded in Uniform Manifold Approximation and Projection (UMAP) space (left: true labels; right: predicted labels) using Seurat (25) based on 20 PCs calculated from 500 HVGs. The quality of classification was evaluated using Matthew’s correlation coefficient (MCC), which was calculated for the initial labels, for a baseline model using logistic regression on the initial labels and for optimization of the initial labels using valiDrops. For sensitivity analysis, we used a stratified subsampling approach. Barcodes were grouped into 10 groups based on the score used as the basis for the initial labels, and between 10% and 50% of truly dead barcodes were randomly selected and removed in each group. Each subsampling was repeated 20 times.

Two datasets that had not been analysed as part of the creation of the method were used to validate the method. First, an unsorted sample from the Ordoñez-Rueda datasets (35) was quality filtered using valiDrops with default parameters. Barcodes that pass quality control were combined with barcodes from the sorted live and sorted dead cells. The first 10 PCs based on 100 HVGs were calculated using Seurat (25) and subsequently used for nearest neighbour analysis using RANN. Transcriptomic similarity on pseudo-bulk expression levels was measured by the normalized RMSE (NRMSE) on the top 100 most highly expressed genes. The RMSE was normalized by the interquartile range in the reference samples. Second, raw sequencing data from public scRNA-seq data from the spleen, lungs and oesophagus either immediately processed (fresh) or stored for 72 h prior to processing (36) were downloaded from ENA under accession number PRJEB31843, aligned to the human genome version hg38 using STARsolo (29) and analysed using valiDrops with default parameters. Initial cell type labels were transferred from annotations by the original authors. Barcodes that passed quality filtering in valiDrops, but not in the original analysis, had no labels. For these barcodes, labels were labelled using majority voting in a *k*-nearest neighbour classifier using the 10 nearest neighbours in the 20 first PCs calculated from 2000 HVGs. Ties were broken by selecting the label with the smallest median Euclidean distance. Dead cell labels were only used for runs marked as successful by valiDrops. All barcodes in unsuccessful runs were labelled as live. Transcriptomic similarity on pseudo-bulk expression levels was measured per cell type by the NRMSE on the top 100 most highly expressed genes on subsamples of either fresh cells or cells predicted to be either live or dead after 72 h of storage. The RMSE was normalized by the interquartile range in the fresh cells. For each cell type, we matched group sizes by randomly selecting (with replacement) the same number for each group.

We interpreted how the algorithm makes decisions by extracting the final coefficients from the glmnet models and weighting them by the standard deviation of the input data using the XYZ method. The absolute feature loadings from the singular value decomposition were multiplied by the weighted coefficients and summarized across all components. The average feature contribution was calculated across all 10 different glmnet models and features with a contribution score above 0.1 were submitted to pathway analysis using the enrichR package and the Gene Ontology Biological Processes 2023 database. Pathways that were significantly enriched in either the O’Flanagan dataset or the Ordoñez-Rueda dataset were kept, and the number of pathways was reduced by calculating the Jaccard distance between the genes in each enriched term and clustering the matrix using hierarchical clustering. For each cluster (consisting of different terms with highly overlapping gene sets), the most strongly enriched term was kept.

## Results

### Overview of methods and the benchmarking strategy

**Figure 1.**
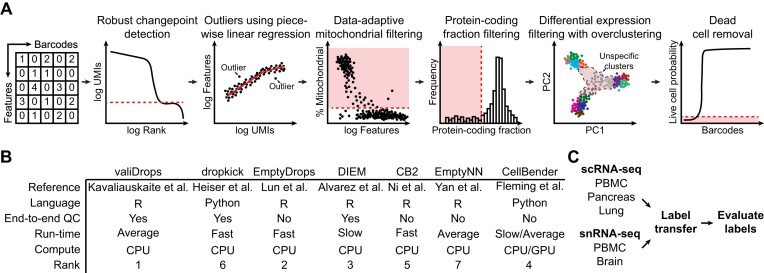
Schematic overview of methods. (**A**) Illustration of the major steps in valiDrops. (**B**) Overview of existing methods in the field, specifying the programming language, whether they require post-filtering, an estimate of their run time, the compute backend they use and the rank they achieved in our benchmarking. CellBender (18) is significantly faster if run using a graphics processing unit. (**C**) Overview of the benchmarking approach and datasets used to valiDrops and benchmark to existing tools. The indicated datasets were quality filtered using valiDrops or alternative methods. Azimuth (25) was used to transfer labels from reference atlases and the resulting transferred labels were evaluated in terms of homogeneity and separability.

valiDrops takes as input an unfiltered feature-by-barcode matrix, which can be produced by all common alignment and count methods, for example CellRanger (10), STARsolo (29), kallisto|bustools (37) and alevin (38), and sequentially removes barcodes of low quality (Figure 1A). Some of the quality metrics used in valiDrops for barcode filtering, such as the total number of UMIs, are already extensively used in the field but often require user input to set thresholds. In contrast, valiDrops automatically detects thresholds, such as the threshold defining the ‘ambient plateau’, and removes barcodes with a low number of UMIs, which are likely to contain only ambient signals. Next, valiDrops uses stepwise linear regression to infer the relationship between the number of detected features and the number of UMIs and automatically removes outliers. It uses knee-point detection to automatically detect an appropriate threshold for filtering based on the fraction of UMIs derived from mitochondrial genes, and it fits a normal distribution to the fraction of UMIs derived from protein-coding genes and sets a threshold using the fitted mean and standard deviation. In addition to these commonly used quality metrics, valiDrops uses a novel filtering approach based on differentially expressed genes. Barcodes are grouped into both high-resolution and low-resolution clusters using a combination of graph clustering and a data-adaptive method for selecting clustering resolutions. The barcodes in each high-resolution cluster are then compared to all other barcodes, which do not belong to the same low-resolution cluster, and any high-resolution clusters that do not show significant differences in gene expression compared to the other barcodes are removed. This process helps to remove droplets containing a cell or nucleus with a low signal-to-noise ratio. Finally, valiDrops has the option to predict and flag putative dead cells. To detect dead cells, valiDrops initially labels dead cells using a simple heuristic based on the fraction of UMIs assigned to mitochondrial, ribosomal and protein-coding genes. Next, valiDrops refines these noisy labels using ridge regression and an adaptive resampling strategy.

Here, we benchmarked valiDrops and six other existing tools and found that valiDrops has the best performance and an average run time (Figure 1B). It is an open problem how to benchmark barcode filtering methods since there are no ground truth datasets available. Here, we devised a strategy based on assessing the quality of transferred labels after barcode filtering (Figure 1C). The use of label transfer circumvents any biases towards methods used by the original authors of benchmarking datasets, thereby preventing any artificial inflation of the performance of mainstream methods. Additionally, any potential biases introduced by label transfer will affect all methods equally ensuring a fair comparison. For each tool, we filtered barcodes across 47 single-cell or single-nucleus RNA-seq samples from five different datasets and subsequently used Azimuth (25) to transfer cell type labels. Then, we assessed the quality of the identified barcodes by evaluating labelling metrics, conservation of label hierarchies, clustering of labels and intra-label transcriptional entropy. Additionally, we integrated samples from the same dataset using Seurat (25), and assessed the extent of batch effects by evaluating the conversation of biological signals, separation of batch labels and similarity of cell type labels using both cluster-dependent and cluster-independent metrics.

### valiDrops sequentially removes low-quality barcodes

**Figure 2.**
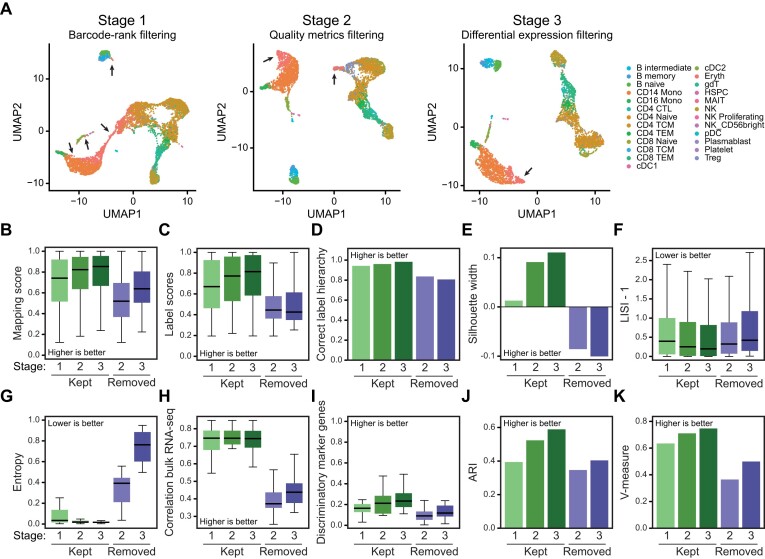
Quality filtering using valiDrops improves data quality. (**A**) Barcodes that pass filtering on total UMI counts (stage 1), filtering on quality metrics (stage 2) and filtering on expression-based metrics (stage 3) from a reference PBMC sample from 10X Genomics (5k PBMC scRNA-seq, Supplementary Table S3) were embedded in the UMAP space using Seurat (25) based on 10 PCs calculated from 2000 HVGs. Labels were transferred using Azimuth (25) with its associated PBMC reference atlas. The arrows point to erythrocytes. (**B**–**K**) Metrics for barcodes that pass filtering in stage 1, 2 or 3, as well as for barcodes that are removed either between stages 1 and 2 or between stages 2 and 3: (**B**) Box plot of mapping scores calculated by Azimuth during label transfer. (**C**) Box plot of label scores calculated by Azimuth during label transfer. (**D**) The fraction of labels that have the correct label hierarchy (**E**) The average silhouette width across transferred labels in the first 10 PCs. (**F**) Median LISI across transferred labels in the first 10 PCs. (**G**) Entropy of the top 200 most highly expressed genes in each label class. (**H**) Box plot of median Spearman’s correlation coefficient between pseudo-bulk expression levels and the expression levels in RNA-seq from sorted cells of the same cell type (39). (**I**) The fraction of marker genes in a label class, defined as differentially expressed genes (FDR ≤ 0.05, log fold change > 0) compared to all other barcodes, with an AUC higher than 0.9. (**J**) Bar plot showing the highest ARI across multiple resolutions of Louvain-based clusters and transferred labels (see the ‘Materials and methods’ section). (**K**) Bar plot showing the highest V-measure across multiple resolutions of Louvain-based clusters and transferred labels (see the ‘Materials and methods’ section).

To evaluate how each stage in valiDrops improves data quality, we analysed a single PBMC dataset from 10X Genomics containing 5000 PBMCs and transferred labels using Azimuth (25) to barcodes passing each of three stages in valiDrops. In stage 1, valiDrops removed barcodes with a low number of UMIs. In stage 2, valiDrops removed barcodes using common quality metrics, such as the number of UMIs derived from mitochondrial genes and the relationship between the number of UMIs and number of detected genes. In stage 3, valiDrops removed barcodes without distinct biological signals using a data-adaptive clustering approach and differential expression testing. After stage 1, most transferred cell type labels are well separated in the UMAP space (Figure 2A, left panel). However, major clusters of monocytes and T cells are connected by putative erythrocytes, which are also found in several places across the embedded space (black arrows). After stage 2, the cell types are clustered more tightly. Most erythrocytes connecting major clusters of monocytes and T cells have been removed, although a tail in both clusters is retained (Figure 2A, middle panel). Finally, after stage 3, the cell types are more well separated in the UMAP space, and the tails of erythrocytes are removed leaving one distinct cluster of putative erythrocytes (Figure 2A, right panel).

To quantify the extent to which each of the stages improved the quality of the dataset, we used a large and diverse panel of measures related to labelling, cell type separability, transcriptome similarity and cell type clusterability (i.e. the precision at which clustering can separate cell type labels) (Figure 2B–K; see the ‘Materials and methods’ section). We find that labelling scores and the accuracy of the label hierarchy improved for each stage and that the removed barcodes have much lower scores than the retained barcodes (Figure 2B–D). To determine the accuracy of the label hierarchy, we labelled the barcodes with both coarse-grained and fine-grained labels and asked how large a fraction of barcodes was assigned to a fine-grained label, which was a child of the assigned coarse-grained label. We found a similar trend across all metrics; cluster separability increased across filtering stages and removed barcodes have low separability (Figure 2E and F). Transcriptomic similarity, correlation to bulk RNA-seq expression using bulk RNA-seq data from sorted cells of the same cell type (39) and the fraction of markers that were discriminatory also increased across filtering stages (Figure 2G–I), and so did the ability to cluster barcodes into cell types (Figure 2J and K). Collectively, this strongly indicates that valiDrops filtering improves the quality of the dataset.

### valiDrops compares favourably to existing tools

To compare valiDrops to existing methods, we ran all methods, except CellBender, using default parameters. CellBender does not have a default parameter set, as it requires users to specify the number of expected cells, as well as the number of total cells. In this benchmark, we derived these numbers from the automatically determined thresholds set by valiDrops. Finally, one of the key distinguishing features of valiDrops is that it is automated, does not require post-filtering and does not require user input. In contrast, EmptyDrops, scCB2 and EmptyNN all require post-filtering to remove barcodes containing low-quality cells or nuclei. To ensure that these tools did not artificially underperform due to a lack of post-filtering, we applied miQC (31) to automatically filter barcodes based on mitochondrial content for the scRNA-seq samples and set a data-driven mitochondrial threshold at three times the median absolute deviation above the median for single-nucleus RNA-seq samples.

**Figure 3.**
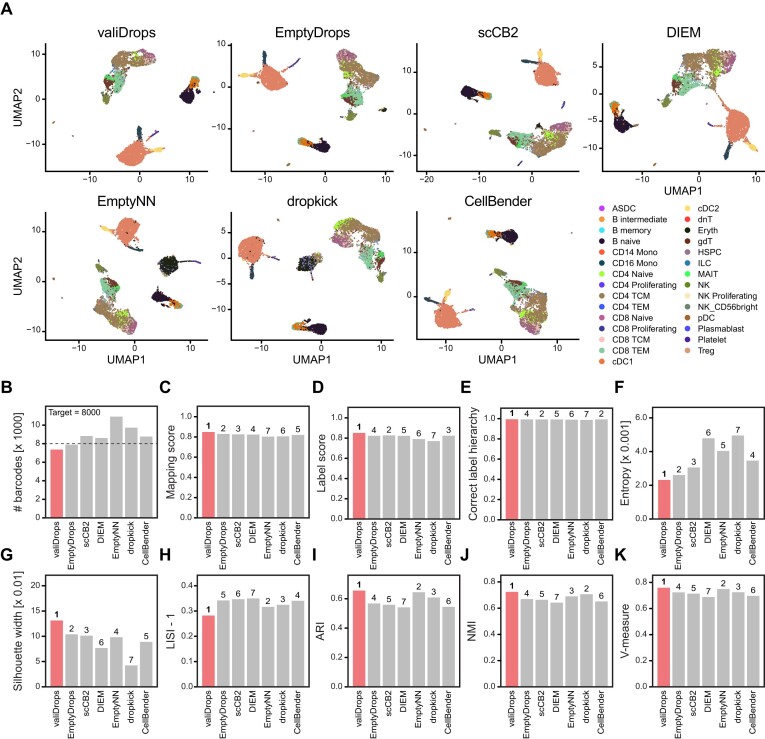
Comparison of valiDrops and alternative quality control methods on PBMC data. (**A**) Barcodes that pass filtering by valiDrops, EmptyDrops (14), DIEM (16), scCB2 (15), dropkick (19), CellBender (18) or EmptyNN (17) from a reference PBMC sample from 10X Genomics (8k PBMC scRNA-seq, Supplementary Table S3) were embedded in the UMAP space using Seurat (25) based on 10 PCs calculated from 2000 HVGs. Labels were transferred using Azimuth (25). (**B**) The number of barcodes that pass filtering in each method. Vertical line: the experimentally targeted number of cells. (**C**–**K**) Metrics for barcodes filtered as in panel (A). The numbers indicate the rank of the method, where 1 is the best and 7 is the worst. valiDrops is highlighted. (**C**) Average label transfer mapping score calculated by Azimuth. (**D**) Average label transfer score calculated by Azimuth. (**E**) The fraction of labels that have the correct label hierarchy (see the ‘Materials and methods’ section). (**F**) Entropy of top 200 most highly expressed genes in each label class. (**G**) Average silhouette width across transferred labels in the first 10 PCs. (**H**) Median LISI across transferred labels in the first 10 PCs. Maximum ARI (**I**), NMI (**J**) or V-measure (**K**) across multiple resolutions of Louvain-based clusters and transferred labels (see the ‘Materials and methods’ section).

Initially, we evaluated a single PBMC dataset from 10X Genomics containing 8000 PBMCs (Figure 3A). All methods detected ∼8000 high-quality barcodes, except dropkick and EmptyNN, both of which detected >9000 barcodes (Figure 3B). Across the labelling metrics, valiDrops achieved the highest mapping score, but scores were overall similar (Figure 3C–E). However, barcodes identified by valiDrops had the highest transcriptome similarity within cell types, as measured by entropy (Figure 3F). For cell type cohesion and clusterability (see the ‘Materials and methods’ section), valiDrops achieved the best scores for the silhouette width, for the LISI (Figure 3G and H) and for all clustering metrics (Figure 3I–K). Thus, in this dataset, valiDrops exhibited the best performance across all metrics.

**Figure 4.**
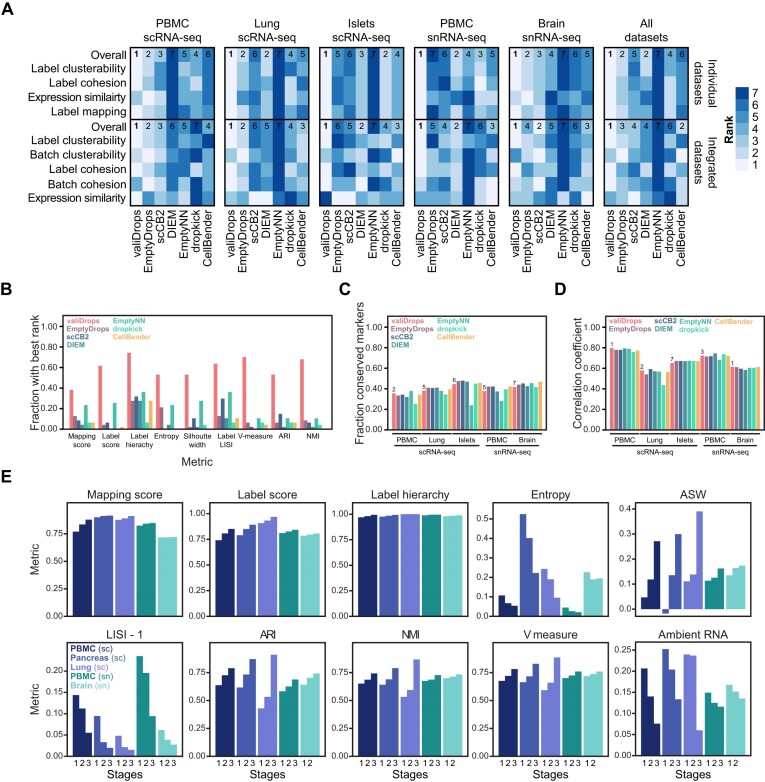
Benchmarking valiDrops and alternative quality control methods. Barcodes were quality filtered using valiDrops, EmptyDrops (14), DIEM (16), scCB2 (15), dropkick (19), CellBender (18) or EmptyNN (17), and labels were transferred with Azimuth (25) with associated reference atlases. The value in each table is the overall rank of the method based on individual dataset metrics, where lower is better (see the ‘Materials and methods’ section for a detailed description of the metrics). For the integration rank, each sample was quality controlled individually and integrated using the RPCA method in Seurat (25) with default parameters. The value in each table is the overall rank of the method based on integration metrics, where lower is better (see the ‘Materials and methods’ section for a detailed description of the metrics). (**A**) Heatmap for ranks for all datasets, as well as an overall rank across all datasets. (**B**) Bar plot showing the fraction of datasets that achieved the best rank for each metric and for each method. (**C**) Bar plot showing the fraction of conserved marker genes across the five integration datasets for each method. (**D**) Bar plot showing the median Spearman’s rank coefficient of correlation between pseudo-bulk expression levels per class label per dataset across the five integration datasets for each method. (**E**) Bar plots showing the average of the indicated metrics (see the ‘Materials and methods’ section for details on their calculation) for the indicated datasets considering barcodes passing stage 1, 2 or 3 of valiDrops.

We expanded this benchmark by analysing a total of 47 sxRNA-seq samples from five datasets. Each method was ranked based on the set of nine metrics (the overall rank), as well as on subsets of metrics that were used to evaluate label mapping, expression similarity, label cohesion and label clusterability (Supplementary Table S1). In addition, we integrated the samples from each dataset using Seurat and evaluated the integration in terms of marker gene conservation, cross-sample gene-rank correlation, integration metrics and biological conservation metrics (Supplementary Table S2). Each method was ranked based on the full set of 12 metrics (the overall rank) and on subsets of metrics, which were used to evaluate expression similarity, label cohesion, label clusterability, batch cohesion and batch clusterability (see the ‘Materials and methods’ section). For all five datasets and integration tasks, valiDrops achieved the best overall rank (Figure 4A). For filtering individual samples, valiDrops achieved the best overall rank for 35 of the 47 samples, rank in the top 2 for 44 of the 47 samples and never ranks worse than 4. This high performance was consistent across all metrics (Figure 4B). For batch integration after filtering, valiDrops achieved the best overall rank for all tasks, but notably did not consistently achieve a high rank for expression similarity. However, looking at the individual metrics that compose this rank, we found that all methods perform approximately similarly (Figure 4C and D). Thus, valiDrops achieves state-of-the-art performance in terms of barcode filtering improving data quality and retaining true biological signals, and dissection of each step in valiDrops revealed that all three stages are required to achieve the best performance across all datasets (Figure 4E).

### valiDrops can detect dead cells with high accuracy

Recent literature suggests that a subset of dead cells can pass regular quality control and confound the transcriptomic signatures of cell types or states in the sxRNA-seq dataset (3,35). This issue may be especially prevalent in certain diseases associated with the induction of cell death, or when storing tissues for later processing, where cell types within the tissue may have a differential sensitivity to the storage conditions.

**Figure 5.**
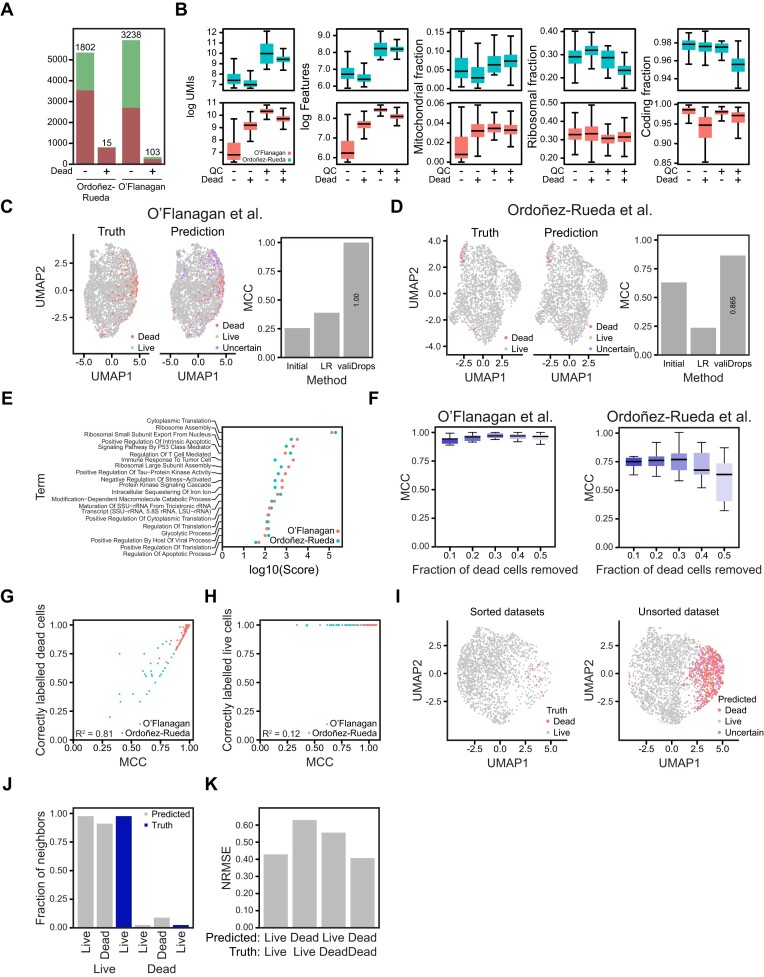
Identification of dead cells using valiDrops. (**A**) Bar plot showing the number of cells passing (green) or failing (red) quality filtering by valiDrops for dead and live cells from the O’Flanagan datasets (3) and the Ordoñez-Rueda datasets (35), respectively. (**B**) Box plots showing the indicated metric for barcodes that either pass or fail quality control (QC) and that are either dead or live for the indicated datasets. (**C**, **D**) Barcodes that pass quality control for the indicated datasets were embedded in the UMAP space (left: true labels; right: predicted labels) using Seurat (25) based on 20 PCs calculated from 500 HVGs. The bar plots show the MCC for the initial labels, for a baseline model using logistic regression on the initial labels (LR) and for valiDrops. (**E**) Scatter plot showing the log_10_ combined score for the indicated datasets of pathways significantly enriched for genes with high feature importance in dead cell prediction. (**F**) Box plots showing MCC for dead cell prediction in the indicated dataset after removal of the indicated fraction of truly dead cells. (**G**) Scatter plot of the MCC and the fraction of truly dead cells that were correctly labelled. (**H**) Scatter plot of the MCC and the fraction of truly live cells that were correctly labelled. (**I**) UMAP embedding (left: sorted datasets with true labels; right: unsorted datasets with predicted labels) using Seurat (25) based on 10 PCs calculated from 100 HVGs of an unsorted sample from the Ordoñez-Rueda dataset (35) after quality filtering and dead cell prediction. (**J**) The fraction of 10 nearest neighbours derived from truly live (first three bars) or truly dead (last three bars) cells computed on the 20 first PCs calculated from 100 HVGs for predicted live, predicted dead or truly live cells. (**K**) The NRMSE between pseudo-bulk expression levels for top 100 most highly expressed genes in truly live cells and either predicted live or predicted dead cells, and between the pseudo-bulk expression levels for top 100 most highly expressed genes in truly dead cells and either predicted live or predicted dead cells.

Re-analysis of these datasets with valiDrops revealed that valiDrops removes a large fraction of dead cells through regular quality control (Figure 5A), but that there are dead cells that pass quality control. The dead cells that pass quality control are characterized by a higher number of UMIs and higher coding fraction than live cells passing quality control, but they are not easily distinguishable based on quality control metrics alone (Figure 5B). To overcome potential confounding from dead cells, we designed an optional module in valiDrops to predict dead and live cells. To create this module, we leveraged two datasets with ground truth, where the authors had induced cell death, sorted cells into live and dead populations, and performed scRNA-seq on both groups (3,35). Based on these labels, we created a module with three steps. Initially, every cell is assigned a score based on arcsine-transformed fractions of UMIs derived from mitochondrial genes, ribosomal genes and coding genes, as well as the standardized number of total UMIs and detected genes. Second, the cells are labelled using a data-adaptive thresholding approach to separate the cells into a group enriched for dead cells and a mixed group containing both live and dead cells. Finally, the labels are optimized using ridge regression and a modified version of adaptive resampling (40).

In the two datasets, initial labels made by valiDrops were weak predictors of the true class, whereas the labels optimized using adaptive resampling and ridge regression were highly accurate (MCC ≥ 0.86), and significantly better than baseline models using logistic regression on the initial labels (Figure 5C and D). For both models, we derived feature importance scores and found that the genes important for prediction are associated with translational processes, stress-related protein kinases and tau-protein kinases, and apoptotic processes (Figure 5E). To evaluate the sensitivity of valiDrops, we used a stratified subsampling approach to approximate the distribution of initial scores but reduce the number of true dead cells by between 10% and 50%. In one dataset, valiDrops maintained a median MCC above 0.9 across the subsampled datasets, while in the other the MCC decreased to a median of ∼0.66 after the removal of 50% of the truly dead cells. Although the MCC decreases, this still represented a strong predictive performance (Figure 5F). The difference in MCC between the datasets was likely explained by the absolute numbers of truly dead cells present after subsampling, as the dataset with the lowest MCC only contained a median of 4 truly dead cells, whereas the dataset with the highest MCC contained a median of 50 truly dead cells. The decrease in MCC observed at small numbers of truly dead cells was driven by a decrease in the ability to correctly label dead cells (Figure 5G), not in the ability to correctly label live cells (Figure 5H). Reassuringly, this highlights that valiDrops does not spuriously flag live cells for removal.

To test the module on unseen data, we processed and predicted dead cells in an additional sample included in one of the ground truth datasets (35), where cell death had been induced, but the cells had not been sorted. To evaluate the predicted labels, we integrated the unsorted and sorted datasets. The predicted dead cells in the unsorted dataset are more often neighbours to truly dead cells than to truly live cells in the integrated reduced dimensional space. Similarly, the predicted live cells are more often neighbours to truly live cells than to truly dead cells (Figure 5I and J). Comparison of the pseudo-bulk transcriptomic profile of predicted live and predicted dead cells to that of truly live and truly dead cells revealed that the predicted live cells are transcriptionally more like truly live cells, while predicted dead cells are more like truly dead cells (Figure 5K). This suggests that *in silico* dead cell labelling can rescue samples that are contaminated with dead cells or serve as a basis for studying mechanisms of cell death.

**Figure 6.**
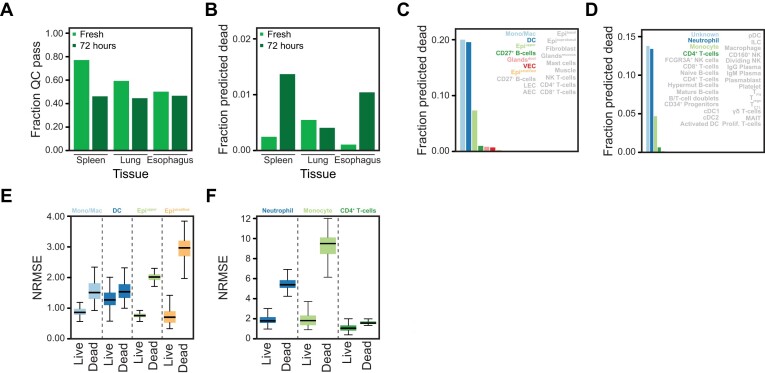
Improving data quality by dead cell prediction. (**A**) Bar plot of the fraction of barcodes that pass filtering for low total UMI counts (stage 1) that pass final quality filtering for fresh samples and samples stored for 72 h from the spleen, lungs and oesophagus (36). (**B**) The fraction of barcodes that pass final quality control that are predicted to be dead. (**C**, **D**) The fraction of predicted dead cells by cell type labels (K: oesophagus; L: spleen). Cell types with >1% dead cells are highlighted. (**E**, **F)** Box plot across subsamples showing NRMSE between pseudo-bulk expression levels for top 100 most highly expressed genes in fresh cells and either predicted live or predicted dead cells stored for 72 h for the indicated cell types (M: oesophagus; N: spleen). Cell types with at least 1% of the cells, and at least a total of five predicted dead cells after storage for 72 h, were included in the analysis.

To test the module in more biologically relevant systems, we re-analysed data from a study that assessed the effects of cold storage of up to 72 h on healthy human spleen, oesophagus and lungs (36). The original authors showed using TUNEL staining that on the tissue level especially the spleen and oesophagus had increased numbers of dead cells after 72 h of cold storage. The spleen and lung samples were processed with a dead cell removal kit, and all three tissues were analysed by scRNA-seq. In the initial quality control using valiDrops, we found that a decreased fraction of barcodes passed quality control after 72 h of storage, and that this was especially pronounced in the spleen (Figure 6A). This observation was also reported in the original paper based on manual quality control. Across the datasets, cell labelling by valiDrops predicts low numbers of dead cells (Figure 6B), consistent with the oesophagus samples having high viability and the lung and spleen samples being filtered using a dead cell removal kit prior to sample preparation. However, valiDrops did predict an increased number of dead cells in the spleen and oesophagus samples after 72 h consistent with the TUNEL staining. The fraction and the total number of predicted dead cells vary across cell types (Figure 6C and D) suggesting that different cell types have different susceptibilities to dying during cold storage. In cell types marked by high rates of predicted dead cells, the cells predicted to be live are transcriptionally more like cells from the same cell type in the fresh samples compared to cells predicted to be dead (Figure 6E and F). Taken together, these results show how valiDrops can accurately identify dead cells from complex tissues and that removing these cells restores the accuracy of aggregated transcriptomic profiles.

## Discussion

Quality control and filtering are the most important pre-processing steps in analysing sxRNA-seq datasets. The most widely used workflows rely on tools to identify barcodes having transcriptomic profiles that do not resemble the aggregated signal from barcodes with low coverage. These barcodes are then filtered to define high-quality barcodes based on global and user-defined thresholds for the number of detected genes, the UMI count and/or the fraction of UMIs derived from mitochondrial genes. The individual researcher can bias this process, it can be very time-consuming and there can be identification biases against the most prevalent cell types whose transcriptome has the strongest resemblance to the ambient profile. To overcome these issues, we have developed valiDrops, which is an automated software that identifies high-quality barcodes from raw sxRNA-seq count matrices.

In valiDrops, barcodes are automatically filtered by using data-adaptive thresholds on the number of detected genes, the number of UMIs, their association with each other, the fraction of UMIs derived from mitochondrial genes and the fraction of UMIs derived from coding genes. Next, valiDrops used an overclustering-based approach to identify small groups of barcodes that have a distinct signal compared to the other barcodes passing initial filtering. Thus, unlike existing methods, valiDrops does not rely on comparison between high-quality barcodes and low-coverage barcodes, thereby avoiding biases from differences in how cell types contribute to the profile of low-coverage barcodes. The overclustering-based approach uses a combination of clustering resolutions to ensure that barcodes containing cells or nuclei from the same or closely related cell types and states are not compared, thereby avoiding potential biases against, for example, differentiating cells. However, since valiDrops looks for distinct signals, valiDrops can only accurately identify high-quality droplets in datasets with biological heterogeneity. Therefore, we do not advise users to apply the quality control and filtering module of valiDrops to datasets derived from, for example, a single, pure cell line. However, in complex samples, such as tissues or whole organisms, valiDrops is a valuable method for the automatic and unbiased identification of high-quality barcodes and achieves better barcode filtering than existing methods. We based our benchmark on labels obtained through label transfer using reference atlases. This has the advantage of removing any biases towards mainstream methods the original authors used to filter the datasets in the original publications but may potentially introduce a new bias from the label transfer procedure itself, as cells might be wrongly classified or scored. However, this new potential bias equally affects all methods reducing the overall bias affecting method comparison.

Prediction of dead cells by valiDrops has the potential to improve data quality by removing technical biases and to unlock the study of cell death-inducing mechanisms, which is relevant in multiple diseases ranging from cancers to metabolic diseases. To assess the ability of valiDrops to improve data quality when faced with dead cells, valiDrops was used to predict dead cell samples with low or high rates of dead cells prior to dead cell removal and scRNA-seq. Consistently, valiDrops detected more dead cells in samples with a priori high rates of cell death, and computational removal of the predicted dead cells improved the transcriptomic similarity between the sample in question and control samples with low rates of dead cells. The sensitivity of dead cell prediction in valiDrops is high, as valiDrops achieves a median MCC ≥ 0.75 in samples with as little as 0.1% dead cells. Below this threshold, valiDrops increasingly misclassifies dead cells as live, but not live cells as dead. Thus, valiDrops does not spuriously remove truly live cells even in the absence of truly dead cells. We have not tested the ability of valiDrops to flag dead cells in single-nucleus RNA-sequencing datasets, but due to systematic differences in the metrics used to initially flag dead cells, we do not advise users to attempt using single-nucleus RNA-seq datasets as input for dead cell removal.

valiDrops is available as an R package that is available from GitHub (www.github.com/madsen-lab/valiDrops) and requires a single line of code to automatically identify barcodes containing high-quality nuclei or (live) cells.

## Supplementary Material

lqad101_Supplemental_Files

## Data Availability

This work uses only publicly available datasets, and all accession numbers are listed in the ‘Materials and methods’ section. The code underlying valiDrops is available as an installable R package from GitHub at www.github.com/madsen-lab/valiDrops and is permanent hosted at Zenodo (DOI: 10.5281/zenodo.10057279).
