# Supplementary material for: Automatic quality control of single-cell and single-nucleus RNA-seq using valiDrops
Source: NAR Genom Bioinform. 2023 Nov 18;5(4):lqad101. doi: 10.1093/nargab/lqad101 (PMC10657416; doi:10.1093/nargab/lqad101)
Supplement: lqad101_Supplemental_Files [file lqad101_supplemental_files.zip › Supplementary_Table_legends.docx]

**Supplementary Table S1**

Ranks and raw metrics for all individual sample tasks and methods in the benchmark. Related to Figure 3.

**Supplementary Table S2**

Ranks and raw metrics for all integration tasks and methods in the benchmark. Related to Figure 3.

**Supplementary Table S3**

Overview of public datasets used with accession numbers or URLs.
